# *catena*-Poly[[[aqua­silver(I)]-μ-1,1′-(butane-1,4-di­yl)di-1*H*-imidazole-κ^2^ *N* ^3^:*N* ^3′^] hemi(biphenyl-4,4′-dicarboxyl­ate) dihydrate]

**DOI:** 10.1107/S1600536809045826

**Published:** 2009-11-07

**Authors:** Zheyu Zhang

**Affiliations:** a Department of Chemistry, Baicheng Normal College, Baicheng 137000, People’s Republic of China

## Abstract

In the title compound, {[Ag(C_10_H_14_N_4_)(H_2_O)](C_14_H_8_O_4_)_0.5_·2H_2_O}_*n*_, the Ag^I^ ion is three-coordinated by two N atoms from two independent 1,1′-(butane-1,4-di­yl)di-1*H*-imidazole (BBI) ligands and one water O atom in a distorted T-shaped coordination geometry. The biphenyl-4,4′-dicarboxyl­ate (BPDC) dianions do not coordinate to Ag^I^ ions but act as counter-ions. The Ag^I^ ions are linked by BBI ligands, forming a zigzag chain. These chains are linked into a two-dimensional supra­molecular architecture by O—H⋯O hydrogen-bonding inter­actions between water mol­ecules and carboxyl­ate O atoms of the BPDC dianions.

## Related literature

For general background to the design and construction of metal-organic frameworks, see: Kitagawa *et al.* (2004 ▶); Ma *et al.* (2009 ▶); Li *et al.* (2005 ▶). For a related structure, see: Ma *et al.* (2005 ▶).
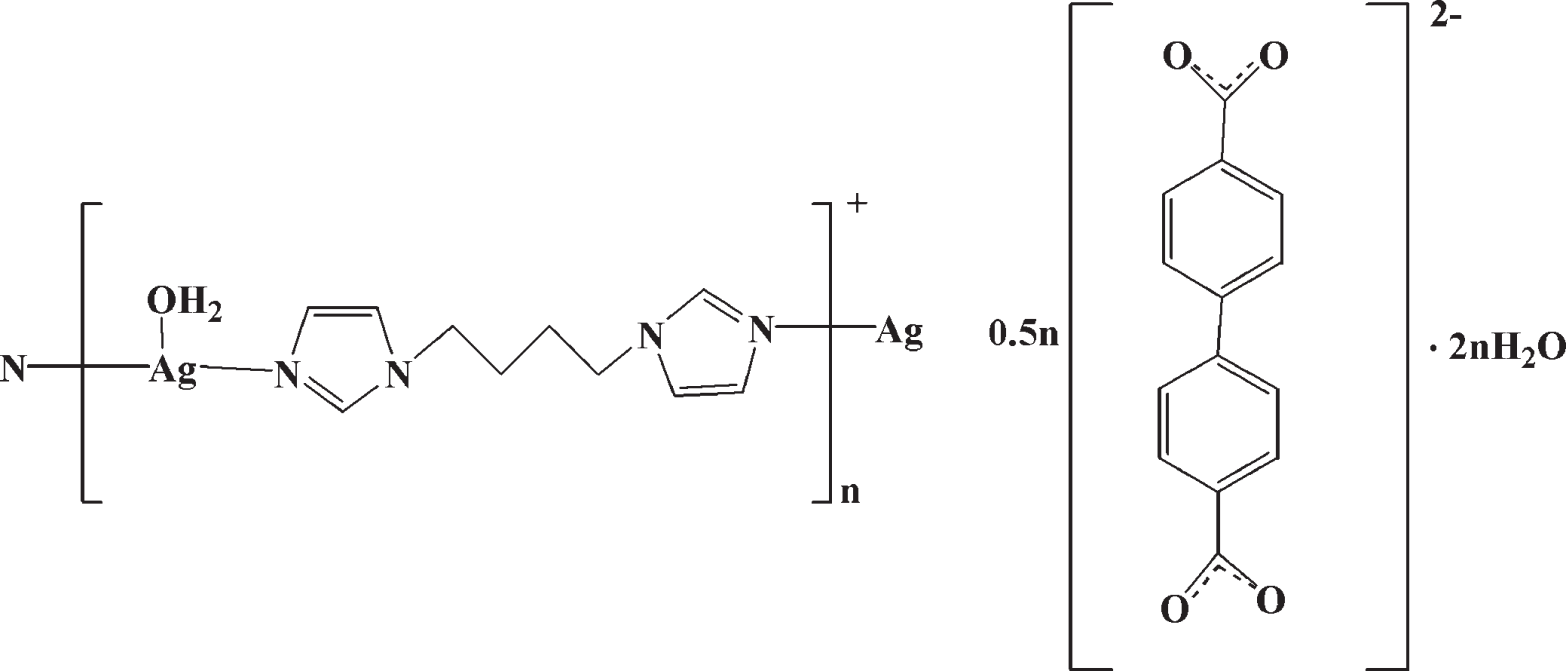


## Experimental

### 

#### Crystal data

[Ag(C_10_H_14_N_4_)(H_2_O)](C_14_H_8_O_4_)_0.5_·2H_2_O
*M* *_r_* = 472.27
Triclinic, 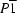

*a* = 9.7685 (6) Å
*b* = 10.0659 (6) Å
*c* = 10.9224 (7) Å
α = 80.190 (1)°
β = 68.898 (1)°
γ = 74.775 (1)°
*V* = 963.36 (10) Å^3^
*Z* = 2
Mo *K*α radiation
μ = 1.08 mm^−1^
*T* = 293 K
0.23 × 0.16 × 0.14 mm

#### Data collection

Bruker APEX CCD area-detector diffractometer
Absorption correction: multi-scan (*SADABS*; Sheldrick, 1996 ▶) *T*_min_ = 0.81, *T*_max_ = 0.86
5289 measured reflections
3569 independent reflections
3422 reflections with *I* > 2σ(*I*)
*R*_int_ = 0.011

#### Refinement

*R*[*F* ^2^ > 2σ(*F* ^2^)] = 0.023
*wR*(*F* ^2^) = 0.058
*S* = 1.06
3569 reflections
267 parameters
9 restraints
H atoms treated by a mixture of independent and constrained refinement
Δρ_max_ = 0.36 e Å^−3^
Δρ_min_ = −0.42 e Å^−3^

### 

Data collection: *SMART* (Bruker, 2002 ▶); cell refinement: *SAINT* (Bruker, 2002 ▶); data reduction: *SAINT*; program(s) used to solve structure: *SHELXS97* (Sheldrick, 2008 ▶); program(s) used to refine structure: *SHELXL97* (Sheldrick, 2008 ▶); molecular graphics: *SHELXTL* (Sheldrick, 2008 ▶); software used to prepare material for publication: *SHELXTL*.

## Supplementary Material

Crystal structure: contains datablocks global, I. DOI: 10.1107/S1600536809045826/ci2938sup1.cif

Structure factors: contains datablocks I. DOI: 10.1107/S1600536809045826/ci2938Isup2.hkl

Additional supplementary materials:  crystallographic information; 3D view; checkCIF report

## Figures and Tables

**Table 1:** Selected bond lengths (Å)

Ag1—N1	2.1209 (17)
Ag1—O1*W*	2.6611 (12)
Ag1—N3	2.1237 (16)

**Table 2:** Hydrogen-bond geometry (Å, °)

*D*—H⋯*A*	*D*—H	H⋯*A*	*D*⋯*A*	*D*—H⋯*A*
O2*W*—H2*A*⋯O1^i^	0.86 (2)	1.99 (2)	2.833 (2)	166 (3)
O2*W*—H2*B*⋯O1^ii^	0.84 (2)	1.95 (2)	2.779 (2)	169 (3)
O3*W*—H3*B*⋯O1^iii^	0.86 (2)	2.05 (2)	2.877 (2)	160 (2)
O3*W*—H3*A*⋯O1*W*	0.86 (2)	2.02 (2)	2.852 (2)	161 (2)
O1*W*—H1*A*⋯O2*W* ^iv^	0.84 (2)	2.03 (2)	2.802 (2)	153 (2)

Symmetry codes: (i) 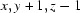; (ii) 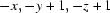; (iii) 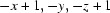; (iv) 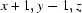.

**Fig. 1.**
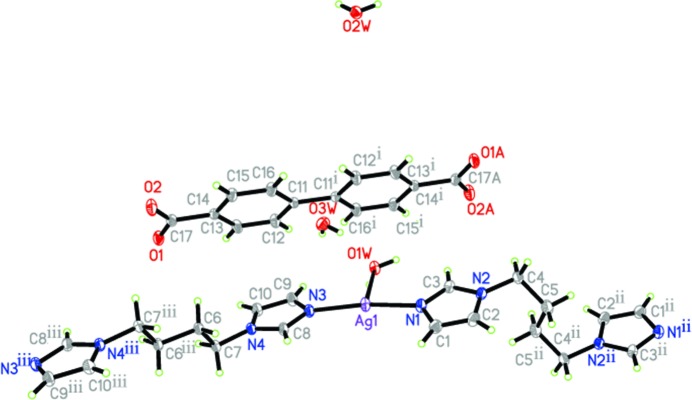
Constituent units of the title compound. Displacement ellipsoids are drawn at the 30% probability level. Symmetry codes: (i) 1 - x, 1 - y, 1 - z; (ii) 3 - x, 1 - y, -z; (iii) 1 - x, -y, 2 - z.

## supplementary crystallographic information

### Comment

Design of effective ligands and the proper choice of metal centers are the keys to design and construct novel metal-organic frameworks (Kitagawa *et al.*, 2004; Ma *et al.*, 2009). These complexes can be specially designed by careful selection of metal cations with preferred coordination geometries, nature of the anions, structures of connecting ligands, and the reaction conditions (Li *et al.*, 2005). In this contribution, we selected biphenyl-4,4'-dicarboxylic acid (H_2_BPDC) as an organic carboxylate anion and 1,1'-(butane-1,4-diyl)di-1*H*-imidazole (BBI) as a N-donor neutral ligand, generating a coordination compound, [Ag(BPDC)_0.5_(H_2_O)(BBI)].2H_2_O, which is reported here.

In the title compound, each Ag^I^ ion is three-coordinated by two N atoms from two independent half-units of the BBI ligands and one water molecule in a distorted T-shaped coordination geometry. The Ag—N and Ag—O distances are comparable to those found in other crystallographically characterized Ag^I^ complexes (Ma *et al.*, 2005). The adjacent Ag^I^ ions are linked by BBI ligands to give a one-dimensional zigzag chain. Biphenyl-4,4'-dicarboxylate anions, acting as counterions, have no contribution to the formation of the final structure (Fig. 1). However, there are intermolecular O—H···O hydrogen bonding interactions among water molecules and BPDC anions. These hydrogen bonds extend zigzag chains into a two-dimensional supramolecular architecture.

### Experimental

To a mixture of biphenyl-4,4'-dicarboxylic acid (0.0484 g, 0.2 mmol) and Ag_2_CO_3_ (0.0275 g, 0.1 mmol) in water was added 1,1'-(butane-1,4-diyl)di-1*H*-imidazole (0.2 mmol, 0.038 g) with constant stirring. After the sample was stirred for 10 min, the precipitate was dissolved by dropwise addition of aqueous NH_3_ solution. Colourless crystals were obtained from the filtrate by slow evaporation after standing in the dark for several days.

### Refinement

Independent atom C5 of the butyl linkage is disordered over two positions with occupancies of 0.852 (8) and 0.148 (8). H atoms of the water molecules were located in a difference Fourier map and refined with an O—H distance restraint of 0.85 (2) Å and with *U*_iso_(H) = 1.5*U*_eq_(O). H atoms on C atoms were generated geometrically and refined as riding atoms with C—H = 0.93 Å and *U*_iso_(H) = 1.2*U*_eq_(C).

### Crystal data

**Table:**

[Ag(C_10_H_14_N_4_)(H_2_O)](C_14_H_8_O_4_)_0.5_·2H_2_O	*Z* = 2
*M**_r_* = 472.27	*F*(000) = 482
Triclinic, *P*1	*D*_x_ = 1.628 Mg m^−^^3^
Hall symbol: -P 1	Mo *K*α radiation, λ = 0.71073 Å
*a* = 9.7685 (6) Å	Cell parameters from 3658 reflections
*b* = 10.0659 (6) Å	θ = 2.0–25.7°
*c* = 10.9224 (7) Å	µ = 1.08 mm^−^^1^
α = 80.190 (1)°	*T* = 293 K
β = 68.898 (1)°	Block, colourless
γ = 74.775 (1)°	0.23 × 0.16 × 0.14 mm
*V* = 963.36 (10) Å^3^	

### Data collection

**Table:**

Bruker APEX CCD area-detector diffractometer	3569 independent reflections
Radiation source: fine-focus sealed tube	3422 reflections with *I* > 2σ(*I*)
graphite	*R*_int_ = 0.011
φ and ω scans	θ_max_ = 25.7°, θ_min_ = 2.0°
Absorption correction: multi-scan (*SADABS*; Sheldrick, 1996)	*h* = −11→11
*T*_min_ = 0.81, *T*_max_ = 0.86	*k* = −12→11
5289 measured reflections	*l* = −12→13

### Refinement

**Table:**

Refinement on *F*^2^	Primary atom site location: structure-invariant direct methods
Least-squares matrix: full	Secondary atom site location: difference Fourier map
*R*[*F*^2^ > 2σ(*F*^2^)] = 0.023	Hydrogen site location: inferred from neighbouring sites
*wR*(*F*^2^) = 0.058	H atoms treated by a mixture of independent and constrained refinement
*S* = 1.06	*w* = 1/[σ^2^(*F*_o_^2^) + (0.0296*P*)^2^ + 0.5838*P*] where *P* = (*F*_o_^2^ + 2*F*_c_^2^)/3
3569 reflections	(Δ/σ)_max_ = 0.003
267 parameters	Δρ_max_ = 0.36 e Å^−^^3^
9 restraints	Δρ_min_ = −0.41 e Å^−^^3^

### Special details

**Table:**

Geometry. All s.u.'s (except the s.u. in the dihedral angle between two l.s. planes) are estimated using the full covariance matrix. The cell s.u.'s are taken into account individually in the estimation of s.u.'s in distances, angles and torsion angles; correlations between s.u.'s in cell parameters are only used when they are defined by crystal symmetry. An approximate (isotropic) treatment of cell s.u.'s is used for estimating s.u.'s involving l.s. planes.
Refinement. Refinement of *F*^2^ against ALL reflections. The weighted *R*-factor *wR* and goodness of fit *S* are based on *F*^2^, conventional *R*-factors *R* are based on *F*, with *F* set to zero for negative *F*^2^. The threshold expression of *F*^2^ > 2σ(*F*^2^) is used only for calculating *R*-factors(gt) *etc*. and is not relevant to the choice of reflections for refinement. *R*-factors based on *F*^2^ are statistically about twice as large as those based on *F*, and *R*- factors based on ALL data will be even larger.

### Fractional atomic coordinates and isotropic or equivalent isotropic displacement parameters (Å^2^)

**Table:**

	*x*	*y*	*z*	*U*_iso_*/*U*_eq_	Occ. (<1)
Ag1	0.956725 (17)	0.248290 (16)	0.414393 (15)	0.03644 (7)	
C1	1.1776 (2)	0.4373 (2)	0.3910 (2)	0.0390 (5)	
H1	1.1738	0.4140	0.4781	0.047*	
C2	1.2573 (2)	0.5253 (2)	0.3053 (2)	0.0390 (5)	
H2	1.3174	0.5731	0.3219	0.047*	
C3	1.1389 (2)	0.4463 (2)	0.2074 (2)	0.0325 (4)	
H3	1.1042	0.4315	0.1431	0.039*	
C4	1.2955 (2)	0.6107 (2)	0.0650 (2)	0.0372 (5)	
H4A	1.2605	0.7083	0.0785	0.045*	0.855 (8)
H4B	1.2593	0.5933	−0.0014	0.045*	0.855 (8)
H4C	1.3203	0.6896	0.0832	0.045*	0.145 (8)
H4D	1.2221	0.6428	0.0209	0.045*	0.145 (8)
C5	1.4677 (3)	0.5751 (3)	0.0147 (3)	0.0374 (9)	0.855 (8)
H5A	1.5036	0.6338	−0.0647	0.045*	0.855 (8)
H5B	1.5038	0.5942	0.0804	0.045*	0.855 (8)
C5A	1.4372 (14)	0.5180 (14)	−0.0276 (12)	0.024 (4)*	0.145 (8)
H5C	1.4122	0.4341	−0.0372	0.029*	0.145 (8)
H5D	1.4686	0.5669	−0.1141	0.029*	0.145 (8)
C6	0.5360 (2)	0.0327 (2)	0.93096 (18)	0.0289 (4)	
H6A	0.4580	0.0804	0.8937	0.035*	
H6B	0.5876	0.1004	0.9370	0.035*	
C7	0.6471 (2)	−0.0754 (2)	0.84084 (19)	0.0305 (4)	
H7A	0.7307	−0.1157	0.8731	0.037*	
H7B	0.5982	−0.1486	0.8433	0.037*	
C8	0.7895 (2)	0.0778 (2)	0.6596 (2)	0.0318 (4)	
H8	0.8211	0.1176	0.7127	0.038*	
C9	0.7547 (2)	0.0266 (2)	0.4916 (2)	0.0357 (5)	
H9	0.7588	0.0248	0.4055	0.043*	
C10	0.6817 (2)	−0.0505 (2)	0.5973 (2)	0.0344 (4)	
H10	0.6266	−0.1132	0.5975	0.041*	
C11	0.4496 (2)	0.46334 (19)	0.55623 (18)	0.0242 (4)	
C12	0.4568 (2)	0.3221 (2)	0.5596 (2)	0.0327 (4)	
H12	0.5256	0.2728	0.4897	0.039*	
C13	0.3640 (2)	0.2543 (2)	0.6645 (2)	0.0326 (4)	
H13	0.3716	0.1603	0.6641	0.039*	
C14	0.2594 (2)	0.32470 (19)	0.77048 (18)	0.0261 (4)	
C15	0.2498 (2)	0.4651 (2)	0.7673 (2)	0.0321 (4)	
H15	0.1797	0.5143	0.8367	0.038*	
C16	0.3426 (2)	0.5328 (2)	0.6629 (2)	0.0318 (4)	
H16	0.3337	0.6270	0.6634	0.038*	
C17	0.1584 (2)	0.2522 (2)	0.88668 (19)	0.0288 (4)	
N1	1.10255 (19)	0.38726 (19)	0.32976 (17)	0.0341 (4)	
N2	1.23205 (18)	0.53009 (17)	0.18881 (17)	0.0315 (4)	
N3	0.82188 (19)	0.10780 (18)	0.53071 (16)	0.0323 (4)	
N4	0.70506 (18)	−0.01762 (17)	0.70361 (15)	0.0286 (3)	
O1	0.17403 (16)	0.12310 (14)	0.88583 (15)	0.0355 (3)	
O2	0.06563 (18)	0.32386 (16)	0.97662 (15)	0.0418 (4)	
O2W	0.08819 (17)	0.93824 (16)	0.11184 (15)	0.0354 (3)	
O3W	0.64273 (19)	0.13798 (18)	0.20741 (18)	0.0471 (4)	
O1W	0.91632 (17)	0.19506 (16)	0.20090 (14)	0.0353 (3)	
H2A	0.128 (3)	0.984 (3)	0.040 (2)	0.053*	
H2B	0.014 (2)	0.908 (3)	0.115 (3)	0.053*	
H3A	0.715 (3)	0.175 (2)	0.202 (3)	0.053*	
H3B	0.677 (3)	0.0563 (19)	0.180 (3)	0.053*	
H1B	0.957 (3)	0.243 (2)	0.130 (2)	0.053*	
H1A	0.958 (3)	0.1105 (17)	0.201 (3)	0.053*	

### Atomic displacement parameters (Å^2^)

**Table:**

	*U*^11^	*U*^22^	*U*^33^	*U*^12^	*U*^13^	*U*^23^
Ag1	0.03500 (10)	0.03866 (11)	0.03170 (10)	−0.01681 (7)	−0.00289 (7)	0.00291 (7)
C1	0.0356 (11)	0.0507 (13)	0.0313 (11)	−0.0134 (10)	−0.0098 (9)	−0.0015 (9)
C2	0.0344 (11)	0.0456 (12)	0.0412 (12)	−0.0159 (9)	−0.0118 (9)	−0.0049 (10)
C3	0.0277 (10)	0.0364 (11)	0.0306 (10)	−0.0102 (8)	−0.0044 (8)	−0.0021 (8)
C4	0.0315 (11)	0.0308 (11)	0.0403 (12)	−0.0079 (9)	−0.0050 (9)	0.0070 (9)
C5	0.0313 (14)	0.0285 (15)	0.0442 (15)	−0.0113 (10)	−0.0033 (11)	0.0055 (11)
C6	0.0295 (10)	0.0319 (10)	0.0240 (10)	−0.0112 (8)	−0.0062 (8)	0.0027 (8)
C7	0.0319 (10)	0.0327 (10)	0.0246 (9)	−0.0115 (8)	−0.0073 (8)	0.0056 (8)
C8	0.0316 (10)	0.0382 (11)	0.0260 (9)	−0.0151 (9)	−0.0069 (8)	0.0019 (8)
C9	0.0406 (12)	0.0438 (12)	0.0240 (10)	−0.0168 (10)	−0.0085 (8)	0.0003 (8)
C10	0.0391 (11)	0.0371 (11)	0.0292 (10)	−0.0153 (9)	−0.0101 (9)	−0.0008 (8)
C11	0.0232 (9)	0.0249 (9)	0.0243 (9)	−0.0071 (7)	−0.0078 (7)	0.0009 (7)
C12	0.0358 (11)	0.0256 (10)	0.0285 (10)	−0.0083 (8)	0.0004 (8)	−0.0030 (8)
C13	0.0384 (11)	0.0230 (9)	0.0322 (10)	−0.0106 (8)	−0.0050 (9)	−0.0003 (8)
C14	0.0241 (9)	0.0294 (10)	0.0258 (9)	−0.0093 (7)	−0.0094 (7)	0.0031 (7)
C15	0.0302 (10)	0.0300 (10)	0.0299 (10)	−0.0076 (8)	−0.0011 (8)	−0.0046 (8)
C16	0.0331 (10)	0.0228 (9)	0.0336 (10)	−0.0090 (8)	−0.0016 (8)	−0.0036 (8)
C17	0.0274 (9)	0.0318 (10)	0.0291 (10)	−0.0123 (8)	−0.0097 (8)	0.0017 (8)
N1	0.0312 (9)	0.0382 (10)	0.0312 (9)	−0.0133 (8)	−0.0053 (7)	−0.0005 (7)
N2	0.0249 (8)	0.0299 (9)	0.0352 (9)	−0.0075 (7)	−0.0049 (7)	−0.0002 (7)
N3	0.0327 (9)	0.0370 (9)	0.0256 (8)	−0.0137 (7)	−0.0062 (7)	0.0032 (7)
N4	0.0294 (8)	0.0310 (8)	0.0237 (8)	−0.0104 (7)	−0.0062 (6)	0.0020 (6)
O1	0.0360 (8)	0.0283 (7)	0.0378 (8)	−0.0146 (6)	−0.0038 (6)	0.0019 (6)
O2	0.0445 (9)	0.0343 (8)	0.0333 (8)	−0.0135 (7)	0.0052 (7)	−0.0010 (6)
O2W	0.0375 (8)	0.0371 (8)	0.0352 (8)	−0.0129 (6)	−0.0157 (7)	0.0029 (6)
O3W	0.0394 (9)	0.0453 (10)	0.0573 (10)	−0.0110 (8)	−0.0152 (8)	−0.0052 (8)
O1W	0.0397 (8)	0.0319 (8)	0.0295 (7)	−0.0129 (6)	−0.0030 (6)	−0.0007 (6)

### Geometric parameters (Å, °)

**Table:**

Ag1—N1	2.1209 (17)	C7—H7B	0.97
Ag1—N3	2.1237 (16)	C8—N3	1.326 (3)
Ag1—O1W	2.6611 (12)	C8—N4	1.344 (3)
C1—C2	1.350 (3)	C8—H8	0.93
C1—N1	1.378 (3)	C9—C10	1.355 (3)
C1—H1	0.93	C9—N3	1.372 (3)
C2—N2	1.371 (3)	C9—H9	0.93
C2—H2	0.93	C10—N4	1.368 (3)
C3—N1	1.328 (3)	C10—H10	0.93
C3—N2	1.338 (3)	C11—C16	1.399 (3)
C3—H3	0.93	C11—C12	1.400 (3)
C4—N2	1.469 (3)	C11—C11^iii^	1.492 (4)
C4—C5	1.531 (3)	C12—C13	1.382 (3)
C4—C5A	1.564 (13)	C12—H12	0.93
C4—H4A	0.97	C13—C14	1.391 (3)
C4—H4B	0.97	C13—H13	0.93
C4—H4C	0.96	C14—C15	1.387 (3)
C4—H4D	0.96	C14—C17	1.509 (3)
C5—C5^i^	1.518 (5)	C15—C16	1.379 (3)
C5—H5A	0.97	C15—H15	0.93
C5—H5B	0.97	C16—H16	0.93
C5A—C5A^i^	1.49 (3)	C17—O2	1.251 (2)
C5A—H5C	0.97	C17—O1	1.269 (2)
C5A—H5D	0.97	O2W—H2A	0.857 (16)
C6—C7	1.519 (3)	O2W—H2B	0.842 (16)
C6—C6^ii^	1.531 (4)	O3W—H3A	0.865 (16)
C6—H6A	0.97	O3W—H3B	0.864 (16)
C6—H6B	0.97	O1W—H1B	0.863 (16)
C7—N4	1.473 (2)	O1W—H1A	0.842 (16)
C7—H7A	0.97		
			
N1—Ag1—N3	169.34 (7)	N4—C7—H7B	109.1
C2—C1—N1	109.61 (19)	C6—C7—H7B	109.1
C2—C1—H1	125.2	H7A—C7—H7B	107.8
N1—C1—H1	125.2	N3—C8—N4	111.21 (18)
C1—C2—N2	106.38 (19)	N3—C8—H8	124.4
C1—C2—H2	126.8	N4—C8—H8	124.4
N2—C2—H2	126.8	C10—C9—N3	109.77 (18)
N1—C3—N2	111.13 (19)	C10—C9—H9	125.1
N1—C3—H3	124.4	N3—C9—H9	125.1
N2—C3—H3	124.4	C9—C10—N4	106.30 (18)
N2—C4—C5	112.46 (18)	C9—C10—H10	126.9
N2—C4—C5A	110.1 (5)	N4—C10—H10	126.9
N2—C4—H4A	109.1	C16—C11—C12	116.87 (17)
C5—C4—H4A	109.1	C16—C11—C11^iii^	121.5 (2)
C5A—C4—H4A	136.1	C12—C11—C11^iii^	121.6 (2)
N2—C4—H4B	109.1	C13—C12—C11	121.43 (18)
C5—C4—H4B	109.1	C13—C12—H12	119.3
C5A—C4—H4B	76.9	C11—C12—H12	119.3
H4A—C4—H4B	107.8	C12—C13—C14	120.93 (18)
N2—C4—H4C	109.8	C12—C13—H13	119.5
C5—C4—H4C	77.1	C14—C13—H13	119.5
C5A—C4—H4C	110.9	C15—C14—C13	118.11 (17)
H4B—C4—H4C	134.0	C15—C14—C17	120.18 (17)
N2—C4—H4D	109.4	C13—C14—C17	121.72 (17)
C5—C4—H4D	132.7	C16—C15—C14	121.05 (18)
C5A—C4—H4D	108.3	C16—C15—H15	119.5
H4A—C4—H4D	75.9	C14—C15—H15	119.5
H4C—C4—H4D	108.3	C15—C16—C11	121.60 (18)
C5^i^—C5—C4	112.6 (3)	C15—C16—H16	119.2
C5^i^—C5—H5A	109.1	C11—C16—H16	119.2
C4—C5—H5A	109.1	O2—C17—O1	124.92 (18)
C5^i^—C5—H5B	109.1	O2—C17—C14	117.50 (17)
C4—C5—H5B	109.1	O1—C17—C14	117.59 (17)
H5A—C5—H5B	107.8	C3—N1—C1	105.41 (17)
C5A^i^—C5A—C4	110.4 (13)	C3—N1—Ag1	127.40 (15)
C5A^i^—C5A—H5C	109.6	C1—N1—Ag1	127.18 (15)
C4—C5A—H5C	109.6	C3—N2—C2	107.47 (17)
C5A^i^—C5A—H5D	109.6	C3—N2—C4	125.54 (19)
C4—C5A—H5D	109.6	C2—N2—C4	126.99 (18)
H5C—C5A—H5D	108.1	C8—N3—C9	105.46 (17)
C7—C6—C6^ii^	111.4 (2)	C8—N3—Ag1	125.56 (14)
C7—C6—H6A	109.4	C9—N3—Ag1	128.95 (14)
C6^ii^—C6—H6A	109.4	C8—N4—C10	107.26 (16)
C7—C6—H6B	109.4	C8—N4—C7	126.55 (17)
C6^ii^—C6—H6B	109.4	C10—N4—C7	126.19 (17)
H6A—C6—H6B	108.0	H2A—O2W—H2B	116 (2)
N4—C7—C6	112.45 (16)	H3A—O3W—H3B	111 (2)
N4—C7—H7A	109.1	H1B—O1W—H1A	114 (2)
C6—C7—H7A	109.1		
			
N1—C1—C2—N2	−0.1 (3)	C2—C1—N1—Ag1	−178.88 (15)
N2—C4—C5—C5^i^	61.3 (4)	N3—Ag1—N1—C3	177.4 (3)
C5A—C4—C5—C5^i^	−32.3 (8)	N3—Ag1—N1—C1	−3.9 (5)
N2—C4—C5A—C5A^i^	−68.5 (14)	N1—C3—N2—C2	−0.1 (2)
C5—C4—C5A—C5A^i^	32.3 (9)	N1—C3—N2—C4	179.42 (18)
C6^ii^—C6—C7—N4	173.50 (19)	C1—C2—N2—C3	0.1 (2)
N3—C9—C10—N4	−0.6 (3)	C1—C2—N2—C4	−179.36 (19)
C16—C11—C12—C13	−1.0 (3)	C5—C4—N2—C3	−121.4 (2)
C11^iii^—C11—C12—C13	179.5 (2)	C5A—C4—N2—C3	−82.8 (6)
C11—C12—C13—C14	0.2 (3)	C5—C4—N2—C2	58.1 (3)
C12—C13—C14—C15	0.7 (3)	C5A—C4—N2—C2	96.6 (6)
C12—C13—C14—C17	−179.23 (19)	N4—C8—N3—C9	−0.2 (2)
C13—C14—C15—C16	−0.8 (3)	N4—C8—N3—Ag1	−178.33 (13)
C17—C14—C15—C16	179.08 (19)	C10—C9—N3—C8	0.5 (3)
C14—C15—C16—C11	0.1 (3)	C10—C9—N3—Ag1	178.52 (15)
C12—C11—C16—C15	0.8 (3)	N1—Ag1—N3—C8	12.2 (5)
C11^iii^—C11—C16—C15	−179.6 (2)	N1—Ag1—N3—C9	−165.4 (3)
C15—C14—C17—O2	1.4 (3)	N3—C8—N4—C10	−0.1 (2)
C13—C14—C17—O2	−178.69 (19)	N3—C8—N4—C7	−179.62 (18)
C15—C14—C17—O1	−178.55 (18)	C9—C10—N4—C8	0.4 (2)
C13—C14—C17—O1	1.3 (3)	C9—C10—N4—C7	179.93 (19)
N2—C3—N1—C1	0.0 (2)	C6—C7—N4—C8	64.4 (3)
N2—C3—N1—Ag1	178.97 (13)	C6—C7—N4—C10	−115.0 (2)
C2—C1—N1—C3	0.1 (3)		

Symmetry codes: (i) −*x*+3, −*y*+1, −*z*; (ii) −*x*+1, −*y*, −*z*+2; (iii) −*x*+1, −*y*+1, −*z*+1.

### Hydrogen-bond geometry (Å, °)

**Table:**

*D*—H···*A*	*D*—H	H···*A*	*D*···*A*	*D*—H···*A*
O2W—H2A···O1^iv^	0.86 (2)	1.99 (2)	2.833 (2)	166 (3)
O2W—H2B···O1^v^	0.84 (2)	1.95 (2)	2.779 (2)	169 (3)
O3W—H3B···O1^vi^	0.86 (2)	2.05 (2)	2.877 (2)	160 (2)
O3W—H3A···O1W	0.86 (2)	2.02 (2)	2.852 (2)	161 (2)
O1W—H1A···O2W^vii^	0.84 (2)	2.03 (2)	2.802 (2)	153 (2)

Symmetry codes: (iv) *x*, *y*+1, *z*−1; (v) −*x*, −*y*+1, −*z*+1; (vi) −*x*+1, −*y*, −*z*+1; (vii) *x*+1, *y*−1, *z*.
